# Immunodominant proteins α-1 giardin and β-giardin are expressed in both assemblages A and B of *Giardia lamblia*

**DOI:** 10.1186/1471-2180-11-233

**Published:** 2011-10-19

**Authors:** Constanza Feliziani, María C Merino, María R Rivero, Ulf Hellman, María C Pistoresi-Palencia, Andrea S Rópolo

**Affiliations:** 1 Laboratorio de Microbiología e Inmunología, Instituto de Investigación Médica Mercedes y Martín Ferreyra, INIMEC - CONICET, Friuli 2434, (5000) Córdoba, Argentina; 2 Ludwig Institute for Cancer Research Ltd, Box 595, SE-751 24 Uppsala, Sweden; 3 Departamento de Bioquímica Clínica, CIBICI-CONICET, Facultad de Ciencias Químicas, Haya de la Torre y Medina Allende, UNC, (5000) Córdoba, Argentina

## Abstract

**Background:**

To date, eight assemblages of *Giardia lamblia *have been described, but only assemblages A and B are known to infect humans. Despite the fact that the genomic, biological, and clinical differences found between these two assemblages has raised the possibility that they may be considered different species, there is relatively limited information on their phenotypic differences. In the present study, we developed monoclonal antibodies against alpha-1 and beta giardin, two immunodominant proteins produced during *G. lamblia *infection, and studied their expression and localization in WB (assemblage A) and GS trophozoites (assemblage B).

**Results:**

The polyclonal antibodies generated against WB trophozoites, particularly those recognizing intracellular proteins as well as the proteins present at the plasma membrane (variable-specific surface proteins), showed cross-reactivity with intracellular proteins in GS trophozoites. The use of monoclonal antibodies against beta giardin indicated ventral disc localization, particularly at the periphery in WB trophozoites. Interestingly, although beta giardin was also restricted to the ventral disc in GS trophozoites, the pattern of localization clearly differed in this assemblage. On the other hand, monoclonal antibodies against alpha-1 giardin showed plasma membrane localization in both assemblages with the bare area of GS trophozoites also being distinguished. Moreover, the same localization at the plasma membrane was observed in Portland-1 (Assemblage A) and in P15 (Assemblage E) trophozoites.

**Conclusions:**

We found differences in localization of the beta giardin protein between assemblages A and B, but the same pattern of localization of alpha-1 giardin in strains from Assemblages A, B and E. These findings reinforce the need for more studies based on phenotypic characteristics in order to disclose how far one assemblage is from the other.

## Background

*Giardia lamblia *is a flagellated unicellular microorganism that causes Giardiasis, a generally self-limited clinical illness [1]. Typically, the infection is characterized by diarrhea, abdominal cramps, bloating, weight loss, and malabsorption, although asymptomatic infection also frequently occurs [2]. *G. lamblia *infection is transmitted by the faecal-oral route and results from the ingestion of cysts through the consumption of contaminated food or water or from person-to-person transmission. *Giardia *is distributed globally and has been detected in nearly all classes of vertebrates, including domestic animals, wildlife and in marine vertebrates [3,4]. Since the 80's, differences have been observed between different isolates of *Giardia*, both in isoenzyme studies and in surface-antigen, as well as in the DNA banding pattern after endonuclease restriction analysis, giving rise to the hypothesis that these differences might explain the various clinical manifestations, host responses and treatment efficacy of human Giardiasis [5-7]. Nowadays, advances in molecular epidemiology have enabled specialized genetic groups (i.e., assemblages) to be identified that are relatively species-specific. Among the eight defined genotypes of *Giardia*, only assemblages A and B are known to infect humans, and these two have shown differences related to axenic *in vitro *culture conditions [8-10], metabolism, biochemistry, DNA content, and clinical features, among others [4,11-13]. All these biological differences may be explained by genetic as well as genomic differences, such as the presence of isolate-specific proteins, unique patterns of allelic sequence divergence, differences in genome synteny and in the promoter region of encystation-specific genes and differences in VSP repertoires [14]. It has, therefore, been suggested that assemblages A and B could be considered to be two different *Giardia *species.

During the vegetative stage of the parasite, the trophozoite attaches to the intestinal microvilli to colonize and to resist peristalsis. The ventral disc allows the parasite to orient, ventral side down, to biological or inert substrates, and is a concave cytoskeletal structure surrounded by a plasma membrane, composed of 3 distinct features (microtubules that are coiled around a bare area; microribbons that protrude into the cytoplasm; and cross-bridges that connect adjacent microtubules) [15]. Three gene families of giardins generally localize to the ventral disc including: (i) annexins (i.e. α-giardins) that are localized at the outer edges of microribbons [16-21]; (ii) striated fiber-assemblins such as β-giardin, which are closely associated with microtubules and δ-giardin (a component of microribbons) [22,23]; and (iii) γ-giardin, which is also a microribbon protein [24].

Alpha-giardins form a large class of proteins encoded by 21 different genes (named α-1 to α-19). All of these 21 alpha-giardin genes in WB were found to be conserved in GS along with the genome synteny, although the structural protein alpha-2 giardin was postulated to be an assemblage A-specific protein of human infective *G. lamblia *[25]. However, in a recent study, Franzén et al. encountered a α-2 giardin-like gene in the assemblage B GS strain, with a 92% aa identity in a syntenic position [14]. Differences occurring in the structural proteins may explain the differences observed in key infection processes such as adhesion and motility between both assemblages.

To date, the intracellular localization of giardins in *G. lamblia *has been performed using rabbit polyclonal antisera or by the use of epitope tagged α-giardins [19,26]. However, both these methods have limitations when attempting to study assemblages A and B because polyclonal antibodies have shown cross-reaction with other proteins, while transfection experiments are difficult to carry out on GS assemblages [27]. Therefore, we developed monoclonal antibodies (mAbs) against the two immunodominant proteins, α-1 giardin and β-giardin, and compared the expression and intracellular localization of these structural proteins in assemblages A and B.

## Methods

### Parasites, cells and media

*G. lamblia *strains WB (American Type Culture Collection 50582); WB clone A6 (American Type Culture Collection 50583); WB clone C6 (American Type Culture Collection 50803); Portland-1 (American Type Culture Collection 30888); P15 (isolated from a pig) and GS trophozoites (American Type Culture Collection 50580), were axenically cultivated in screw cap borosilicate glass tubes in modified TYI-S-33 medium enriched with 10% heat-inactivated fetal bovine serum [28] at pH 7.5 supplemented with 0.1% bovine bile [29] for 72 hours at 37°C. Cultures were harvested by chilling on ice followed by agitation to dislodge attached cells. Trophozoites were collected by centrifugation at 500 × g for 10 min at 4°C and washed three times with PBS. The mouse myeloma cell line NSO (ECACC85110503) was grown in RPMI 1640 (GIBCO) supplemented with 10% fetal bovine serum.

### Mice

Purebred female BALB/c mice (aged 10-12 weeks) were purchased from the Facultad de Ciencias Veterinarias, Universidad de La Plata, and housed at the vivarium of the Instituto Mercedes & Martín Ferreyra (INIMEC-CONICET). They were maintained in our animal facilities, which meet the conditions of the Guide to the Care and Use of Experimental Animals, published by the Canadian Council on Animal Care (with the assurance number A5802-01 being assigned by the Office of Laboratory Animal Welfare (NIH)). Our Institutional Experimentation Animal Committee also approved the animal handling and experimental procedures.

### Antigen preparation

WB *Giardia *trophozoites were harvested, homogenized, and resuspended in 1.0 ml of 250 mM sucrose containing the Complete Protease Inhibitor Cocktail (Roche). The lysate was then sonicated three times at 4°C (30 s, 20 A, in a VCX 130 Sonic Disruptor) and centrifuged at 1,000 × g for 10 min to remove unbroken cells and nuclei. Centrifugal forces of 1,000 × g (P1), 20,000 × g (P2), and 105,000 × g (P3) were then layered on a discontinuous sucrose gradient that was formed by layering 750 μl of 60, 55, 50, 45, 40, 35, 30, and 25% (w/w) sucrose into an SW 40 polyallomer centrifuge tube. The gradient was centrifuged for 18 h at 100,000 × g and fractionated from the top into 7 fractions (named a-g). Proteins were precipitated by the addition of 10% TCA. A 20 μl aliquot from each fraction was analyzed by dot-blotting, using anti-VSP9B10 mAb to detect surface localization, and monoclonal anti-α-tubulin (Sigma, St. Louis, MO) to detect the cytoskeletal fraction.

### Monoclonal antibody production

The P1a to P1c fractions were collected and used as antigen for mouse immunization and monoclonal antibody production. Three female BALB/c mice were subcutaneously injected with 100 μg of antigen emulsified with TiterMax Gold Adjuvant (Sigma, St. Louis, MO) (1:1) on days 1 and 15. On day 30, mice were boosted intravenously with 100 μg of the antigen in PBS. The mouse myeloma cell line NSO was used for fusion with spleen cells obtained from immunized mice. Antibody-secreting hybridomas were screened by indirect immunofluorescence and dot-blotting, using non-encysting WB trophozoites. Several monoclonal antibodies were obtained against different *Giardia *antigens. They were then grown, screened and finally cloned.

### Immunofluorescence

Cells were washed with PBSm (1% growth medium in PBS, pH 7.4), allowed to attach to multi-well slides in a humidified chamber at 37°C for an hour, and the wells were fixed for 30 min with acetone/methanol (1:1) at -20°C. After rehydrating with PBS, the cells were blocked with blocking buffer (3% bovine serum albumin, BSA) in PBS for 30 min, followed by incubation with polyclonal serum (1/100) or undiluted hybridoma supernatant at 37°C for an hour. After washing three times with PBS, the cells were incubated for 1 h in the dark with FITC-conjugated goat anti-mouse secondary antibody (Cappel, Laboratories). Finally, preparations were washed and mounted in Vectashield mounting media. Fluorescence staining was visualized by using a conventional (Zeiss Pascal) inverted confocal microscope, using 100× oil immersion objectives (NA 1.32, zoom X). Differential interference contrast images were collected simultaneously with fluorescence images by the use of a transmitted light detector. Images were processed using FV10-ASW 1.4 Viewer and Adobe Photoshop 8.0 (Adobe Systems) software.

Immunofluorescence in non-permeabilized trophozoites was carried out on live cells. To reduce the background, trophozoites were first incubated with 1% bovine serum in PBSm at room temperature for 1 h. After washing, cells were incubated with 100 μl of undiluted hybridoma supernatant for 1 h at 37°C and then washed 3 times. The cells were incubated with 1:200 dilution of FITC-conjugated goat anti-mouse secondary antibody (Cappel, Laboratories) for 1 h at 37°C. The fluorescence was examined with a Zeiss inverted confocal microscope and analyzed as described above.

### Immunoblotting

For Western blotting assays, parasite lysates were incubated with sample buffer with or without β-mercaptoethanol, boiled for 10 min, and separated in 10% Bis-Tris gels using a Mini Protean II electrophoresis unit (Bio-Rad). Samples were transferred to nitrocellulose membranes, blocked with 5% skimmed milk and 0.1% Tween 20 in TBS, and then incubated with hybridoma supernatants or polyclonal antibodies (1:200) for an hour. After washing 3 times with 0.1% Tween 20 in TBS, the strips were incubated for 1 h with horseradish peroxidase-conjugated polyclonal goat anti-mouse Igs (Dako) and then visualized with autoradiography. Controls included the omission of the primary antibody and the use of an unrelated antibody.

### Immunoprecipitation

*G. lamblia *trophozoites were disrupted in lysis buffer (50 mM Tris, pH 8.0, 120 mM NaCl, 5 mM EDTA, 1% Triton X-100, and protease inhibitors) for 30 min on ice and centrifuged at 13,000 g for 5 min at 4°C. The cell lysate was precleared by using protein A/G-Sepharose beads (Santa Cruz Biotechnology, Santa Cruz, CA) for 30 min at 4°C, and then subsequently subjected to immunoprecipitation by using 300 μl of monoclonal antibodies (G3G10 and 12G5). After incubation overnight at 4°C, protein A/G Sepharose was added, and the incubation was continued for 4 h. The immunoprecipitates were washed three times in lysis buffer and analyzed by SDS-PAGE, stained with Coomassie G-250. The bands detected were cut out and submitted for mass spectrometric analysis.

### In-gel digestion and mass spectrometry

The stained gel bands chosen were treated for in-gel digestion as described [30]. Briefly, the bands were destained with acetonitrile and ammonium bicarbonate buffer, and trypsin (porcine, modified, sequence grade, Promega, Madison, WI USA) was introduced to the dried gel pieces. After overnight tryptic digestion, the peptides from the weaker stained bands were bound to a C18 μZipTip and after washing, eluted with acetonitrile containing matrix (alfa-cyano 4-hydroxy cinnamic acid) directly onto the target plate. The mass lists were generated by MALDI-TOF mass spectrometry on an Ultraflex I TOF/TOF from Bruker Daltonics, Bremen, Germany. The search for identity was performed by scanning the NCBInr sequence database with the tryptic peptides using the current version of the search engine ProFound (http://prowl.rockefeller.edu/prowl-cgi/profound.exe). The spectrum was internally calibrated using autolytic tryptic peptides, and the error was set at +/- 0.03 Da. One missed cleavage was allowed, and methionine could be oxidized. The significance of the identity was judged from the search engine's scoring system and other parameters from the similarity between empiric and calculated peptide masses.

### In vitro adhesion assay

WB and GS *Giardia *trophozoites were grown in complete medium, washed with PBS, and counted. Assays were performed in triplicate in 48-well microtitre plates maintained anaerobically. Each well contained 40,000 trophozoites in 200 μl of complete medium and 2 μl of mAbs (1:20). mAb against VSPs (12C2) was used as a positive control of detachment and agglutination, and anti-HA mAb (non-related antibody) was used as a negative control. All antibodies were heated at 56°C for 40 min to eliminate complement-mediated cytotoxicity. The effects of the antibody were recorded by an observer unaware of the contents, immediately after addition of the reagents (0 h), at 2 h and 4 h. Attached trophozoites were enumerated by phase contrast microscopy using an Olympus microscope, by counting total attached trophozoites in at least 10 random lengthwise scans of each culture well, using a 40× objective. Agglutination was evaluated by counting the number of clusters observed in each culture well, using the 10× objective. Viability of the trophozoites after treatment was evaluated, leaving the cultures for ten days and analyzing the adherent living cells. Descriptive statistics included the calculation of the means and S.D. of the control and experimental groups. Average counts were compared between Ab treatments for statistical differences using the independent samples Student's t-test from the SPSS Statistic program.

## Results and discussion

### Polyclonal antibodies against WB trophozoites are also reactive against GS trophozoites

**Figure 1:**
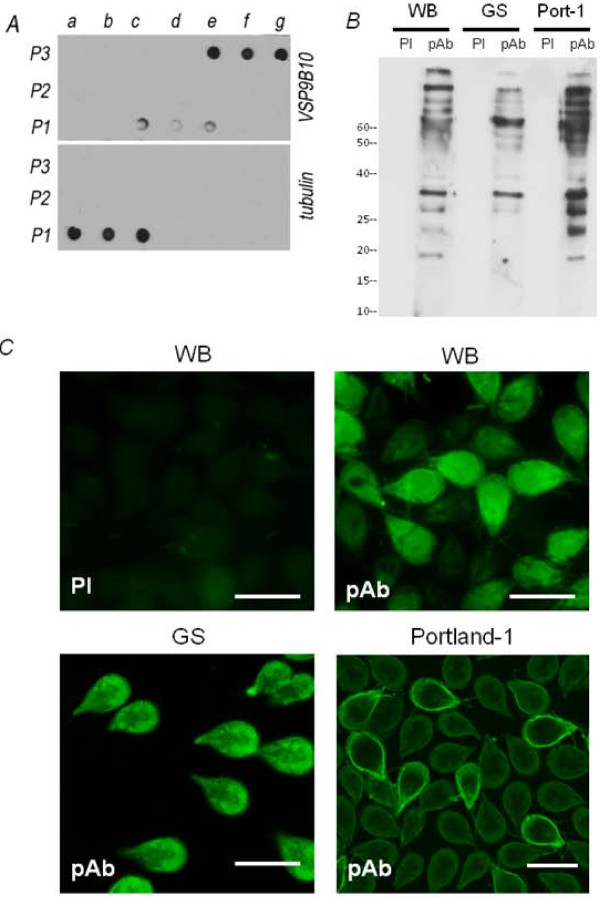
**Polyclonal antibody production**. (A) Dot-blotting of the subcellular fractionation of WB trophozoites shows that surface proteins localized mainly in fractions P3 (samples e-g) and weakly in fraction P1 (samples c-e), while cytoskeleton proteins were found in P1 (samples a-c). P1, P2, and P3 corresponded to the fractions of pellet centrifuged at 1,000 × g, 20,000 × g, and 105,000 × g, respectively. (B) Antibody reactivity. Western blotting of a total WB, GS and Portland-1 *Giardia *lysate incubated with the pre-immune (PI) or the immune polyclonal (pAb) serum. Lane 1: standards of the indicated molecular weights. (C) Reactivity of polyclonal antibodies determined by indirect immunofluorescence in WB, GS and Portland-1 trophozoites. PI: control with pre-immune serum. Scale bar: 10 μm.

Antibodies against variable specific-surface proteins (VSPs) as well as metabolic enzymes were found in patients infected with *Giardia *in both an endemic region (León, Nicaragua) and in a non-endemic area during a waterborne outbreak (Sälen, Sweden). There was also strong immunoreaction to antigens associated with the cytoskeleton, including giardins [31,32]. Therefore, to produce mAbs against giardins, we purified a fraction enriched in cytoskeletal proteins from a lysate of *G. lamblia *trophozoites of the WB strain. After subcellular fractionation, each fraction was analyzed, using mAbs against VSP9B10 (non-cytoskeletal proteins) and tubulin (cytoskeletal protein), by dot-blotting (Figure 1A). The VSP9B10 mAb recognized a VSP that is expressed in WB trophozites, labeling the surface of the trophozoites, including the flagella [33]. The P1a to P1c fractions were collected, and used as the antigen for mouse immunization.

The screening of the polyclonal serum was performed by Western blot and immunofluorescence, in *G. lamblia *WB and Portland-1(assemblage A) and GS (assemblage B) trophozoites. Western blotting showed several bands in WB and Portland-1, but fewer in GS trophozoites (Figure 1B), with the main band of about 30 kDa found in all samples possibly representing the common immunoreactive protein that has been repeatedly identified in natural *Giardia *infections [18,34-36]. By immunofluorescence, we found antibodies reacting against surface proteins in WB as well as in Portland-1, giving uniform staining of the cell surface and flagella, showing a VSP pattern, and against proteins located in distinct subcellular compartments (Figure 1C). Conversely, while these pAbs recognized proteins from diverse subcellular compartments in GS, neither surface proteins nor proteins with a VSP pattern were detected (Figure 1C). Besides the data related to phenotypic similarities or differences between both assemblages, it has been shown at the molecular level that there are only a few assemblage-specific genes, except for the VSP gene family, where the repertoires of the two isolates are completely different [14]. Therefore, it was not surprising that, after immunization with the WB isolate, we found no VSP labeling in GS trophozoites.

The fact that giardins are proteins of approximately 30 kDa, and taking into account their high immunoreactivity, prompted us to analyze whether the production of mAbs against giardins might have resulted from these infected mice. Thus, after fusion, antibody-producing hybridoma cells were selected by immunofluorescence and dot-blotting assays using WB trophozoites. Several antibodies against the ventral disc and the plasma membrane were produced, with the ones that showed immunoreactivity in the immunofluorescence and dot-blotting assays being selected for further analysis. Finally, selected hybridomas were grown, screened and cloned. No typical VSP pattern reactivity was found in GS isolates when they were tested using VSP specific mAb (not shown). Thus, the mAbs that recognized VSPs in WB were not investigated any further.

### Characterization of anti-giardin mAbs

**Figure 2:**
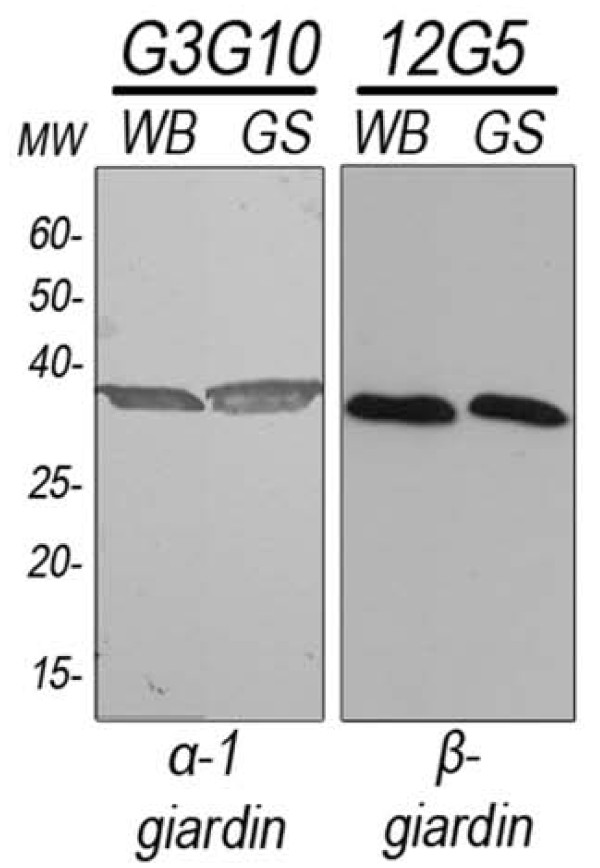
**Western blot analysis of WB and GS *Giardia *proteins recognized by G3G10 (α-1 giardin) and 12G5 (β-giardin) mAbs**. Nitrocellulose membranes were incubated with mAbs and developed with peroxidase-coupled anti-mouse Igs. Lane 1: standards of the indicated molecular weight.

Most giardins showed a plasma membrane localization, with some of these being localized in the ventral disc, and the molecular mass of 30 kDa being a feature of all of them [18,34-36]. Therefore, we selected the monoclonal antibodies that recognized the plasma membrane or ventral disc but also showed a 30 kDa strip in Western blot assays. Among these, G3G10 and the 12G5 mAbs showed reactivity in both WB and GS trophozoites by Western blot assay (Figure 2). The mobility of the 30 kDa protein on SDS-PAGE was the same under either reducing or non-reducing conditions, indicating that it is a single chain protein with few, if any, intrachain disulfide bonds susceptible to reducing agents (data not shown). Immunoprecipitation assays and peptide mass fingerprinting by MALDI-ToF-MS showed that G3G10 mAb recognized α-1 giardin, whereas 12G5 MAb recognized β-giardin in *G. lamblia *(Table 1).

**Table 1:** Mass spectrometry data

EMPIRIC		IN SILICA		PROTEIN IDENTITY	Acc #	**Seq**. **Cov**.	# pep
				
PI	MW	PI	MW				
--	30	5.1	24	Beta-giardin	AAU95567	37	9/40

--	35	6.3	34	Alpha-1 giardin	PI7063	42	12/54

### Differential cellular localization of β-giardin in WB and GS trophozoites

**Figure 3:**
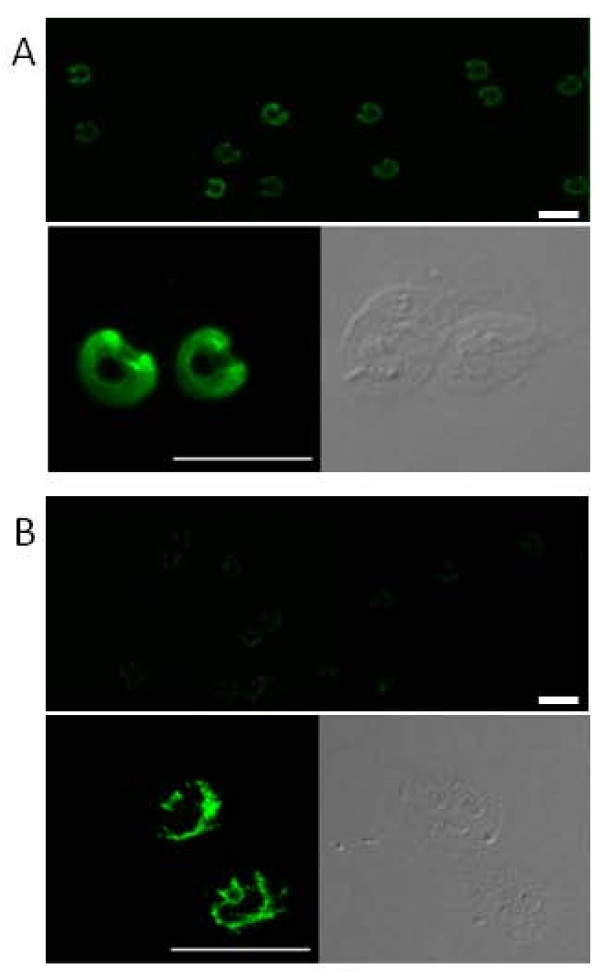
**Immunolocalization of β-giardin in WB and GS trophozoites**. Reactivity of 12G5 mAb on WB and GS *Giardia *trophozoites was determined by indirect immunofluorescence in permeabilized trophozoites. (A) Upper panel: immunofluorescence assays showing the labelling in the ventral disc of the trophozoites. Lower panels: high magnification showing the immunostaining in the ventral disc of WB trophozoites, with more intensity on the margins. (B) Upper panel: immunofluorescence of β-giardin in GS trophozoites. Lower panels: high magnification showing immunofluorescence specifically organized into a radial array surrounding the half ring of the ventral disc and also at the centre of it. Scale bar: 10 μm.

In WB trophozoites, β-giardins assemble in 2.5 nm filaments, which are then further assembled into the superstructure of the dorsal ribbons of the ventral disc, suggesting a primarily structural role for the protein [37,38]. The structural appearance of adhesive discs is essentially identical, not only for different *G. lamblia *assemblages but also for other species such as *G. muris *[37,39,40]. Immunofluorescence assays using anti-β giardin mAb and confocal microscopy showed that β-giardin localized in the ventral disc of WB permeabilized trophozoites (Figure 3A). We have extended the analysis to other Assemblages A isolates (WB clone A6 and Portland-1) and we found no differences with the localization seen in WB 1267 trophozoites (data not shown). The distinctive fluorescence intensity detected at the margins of the ventral disc has been previously reported in *Giardia *trophozoites transfected with GFP-tagged β-giardin or using polyclonal antibodies [41,42]. Some authors have suggested that β-giardin also localizes in the median body of WB trophozoites [43]. However, we did not observe any labeling of the median body, although a large population of trophozoites was analyzed. These differences in localization may suggest that it could be modified, taking into account that Palm et al. found three isoforms of this protein in a proteomic assay [23]. Interestingly, the immunolocalization of β-giardin at the ventral disc in GS trophozoites was rather different, with β-giardin being specifically organized into a radial array that surrounded the half ring of the ventral disc, resembling a horseshoe (Figure 3B). Also, at the center of the ventral disc, an asymmetrical grid could be observed.

The singular localization of β-giardin in WB and GS trophozoites was unexpected, considering that the amino acid sequence of β-giardin is 100% identical in the two assemblages (Additional File 1). Complementary assays utilizing non-permeabilized WB or GS trophozoites showed no fluorescence, showing intracellular β-giardin localization. Related to this, in studies performed on *G. muris *trophozoites, β-giardin was described as a surface protein, based on surface protein biotinilation assays [44]. However, further analysis needs to be performed in order to clarify this point and to attempt to disclose whether the localization of this key protein accounts for the differences in growth and infectivity observed between the two assemblages.

### Expression of α-1 giardin in WB and GS trophozoites

**Figure 4:**
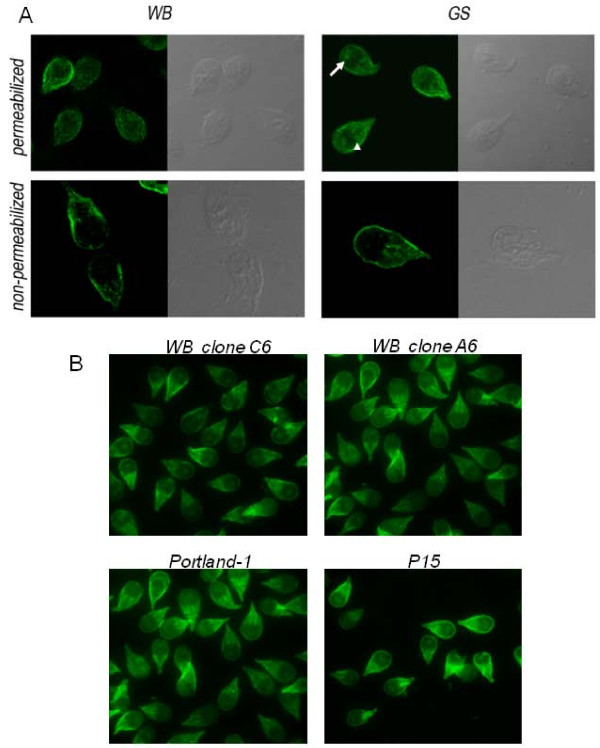
**Immunolocalization of α-1 giardin *Giardia *trophozoites**. (A) Reactivity of G3G10 mAb on WB and GS *Giardia *trophozoites was determined by indirect immunofluorescence in permeabilized (upper panels) and non-permeabilized (lower panels) trophozoites. The arrowheads show the paraflagellar dense rods and the arrows indicate the bare area. Scale bar: 10 μm. (B) Reactivity of G3G10 in permeabilized trophozoites of WB clone C6, WB clone A6, Portland-1 and P-15 strains. Scale bar: 10 μm.

Although earlier studies localized α-1 giardin at the outer edges of the microribbons of the ventral disc in WB trophozoites [40,45], we observed α-1 giardin at the plasma membrane in these cells (Figure 4A). These results are consistent with those observed using a purified pAb against an immunodominant region of α-1 giardin or the AU-1 tagged α-1 giardin transfected trophozoites [19]. An assessment of α-1 giardin localization in the GS strain showed this protein to occur at the plasma membrane as well. Also, α-1 giardin was present in a circular area of vesicles called "the bare area" and also probably in the paraflagellar dense rods, which accompany only the intracellular portions of the corresponding axonemes [46]. Although the differential pattern of localization of α-1 giardin in both strains suggests an additional function of this protein in the B assemblage, supplementary data is still needed in order to reveal if there is a differential function of α-1 giardin in the GS trophozoites.

It has been previously suggested that the localization of α-1 giardin at the plasma membrane, as well as its glycosaminoglycan-binding activity, might be involved in the process by which the parasite binds to the intestinal epithelial cells, an event strongly related to virulence [19]. In the present study, confirmation of the surface expression of α-1 giardin in WB and GS trophozoites was carried out by performing IFA, using non-permeabilized cells (Figure 4A).

Next, we considered the possibility that the presence of α-1 giardin at the plasma membrane may be involved in surface attachment, as was previously demonstrated for δ-giardin [22]. Thus, GS and WB trophozoites were preincubated with mAbs against α-1 giardin, and then attachment, morphology, the presence of cell clusters and viability were analyzed. A time-point examination of the attachment was performed, and compared with trophozoites incubated with anti-VSP antibodies or a non-related antibody (positive and negative controls, respectively). Unlike the anti-VSP mAb, the anti-α1 giardin mAb did not show cell cluster formation or changes in the morphology of the WB (Table 2) or GS trophozoites (not shown). Moreover, no differences in attachment were observed, suggesting that although α-1 giardin is expressed on the cell surface, its role is not directly related to trophozoite attachment *in vitro*. Besides, no differences in growing compared with cells without mAbs were observed. Since it was observed that recombinant α-1 giardin was able to bind to the apical surface of epithelial cells, mast cells, and the connective tissue of the human small intestine [19], it is possible that these proteins might contribute to the stabilization of the interaction between the trophozoite and epithelial cells during *Giardia *infection. On the other hand, during excystation, a functional adhesive disc is absent in the excyzoite, and α-1 giardin localizes to the extracellular membrane of the cell [19]. Therefore, it has been suggested that early during *Giardia *infection, at the period of time where the excyzoite needs to attach in order to avoid peristalsis, α-1giardin probably plays a key role [47]. Adhesion assays using the anti α-1 giardin mAb during excystment should be able to clarify the role played by α-1giardin during trophozoite attachment.

**Table 2:** Effect of mAb treatment on *in vitro *attachment and aggregation of WB *Giardia *trophozoites

	Trophozoite adhesion*			Trophozoite aggregation		
	
	0 hours	2 hours	4 hours	0 hours	2 hours	4 hours
**Without mAb**	20 ± 2	19 ± 2	20 ± 2	-	-	-
**Anti-HA-mAb**	20 ± 2	19 ± 2	22 ± 2	-	-	-
**Anti-VSP-mAb**	21 ± 2	15 ± 2	11 ± 2	-	++	++++
**G3G10-mAb**	19 ± 2	20 ± 2	18 ± 2	-	-	-

*values are an average of 10 random vertical scans of well surface.

The dash (-) indicates no effects. (+) indicates between 4-6 clusters of grouped cells. (++) indicates between 8-10 clusters of grouped cells. (+++) indicates between 15-18 clusters of grouped cells. (++++) indicates more than 20 clusters of grouped cells.

Assays were performed in triplicate and scored by persons unaware of the contents of the wells.

In order to extend the analysis to other *Giardia *strains, we studied the localization of α-1 giardin in WB clone C6, WB clone A6, Portland-1 (Assemblage A) and in P15 trophozoites (Assemblage E). Similar to WB1267 and GSH7, high expression of α-1 giardin near the plasma membrane was observed for these clones. Also, in WB clone C6 and in P15 trophozoites, the bare zone was also stained (Figure 4B). The use of α-1 giardin as an immunizing antigen for the development of a *Giardia *vaccine has been suggested because of its surface localization and its presence during natural *Giardia *infections. However, the fact that both WB and GS trophozoites were unaffected after anti α-1 giardin mAb treatment argues against the use of this protein as a vaccine candidate. Nevertheless, the expression of this protein in assemblage A (WB and Portland-1 strains), in Assemblage B (GS strain) and in Assemblage E (P15 strain), and its immunodominance in sera and feces, strengthen its importance for the development of drug targets or new diagnostic kits for *Giardia*sis.

## Conclusions

While the localization and the functional characteristics of giardins have been described in *Giardia lamblia *of assemblage A isolates, there was no information about the localization or function of giardins in assemblage B. By the development of monoclonal antibodies against the two immunodominant proteins α-1 giardin and β-giardin, we were able to observe the intracellular localization of these structural proteins in assemblages A and B. Taking into consideration some genetic studies as well as the biological differences observed between both strain, it had been proposed that both assemblages might correspond to different species [14]. Although some conclusions may be drawn from genotypic analysis, these need to be supported by phenotypic studies. This is particularly clear for β-giardin, a protein that is 100% homologous at the deduced amino acid level, but with a very different pattern of localization between both assemblages. To date, not enough data is available to define them as separate species. Further genome and transcriptome sequencing, phenotypic studies and correlation with clinical symptoms of different strains within an Assemblage may well be the next steps toward determining species in *Giardia*. These findings could contribute to understanding the variations in pathogenesis associated with infections caused by assemblage A and B isolates of this important parasite.

## Competing interests

The authors declare that they have no competing interests.

## Authors' contributions

CF and ASR carried out the experiments related to the development of monoclonal antibodies. CF, MCM and MRR performed most of the immunoassays and participated in editing the manuscript and data analysis. UH carried out mass spectrometry assays. MCP contributed to the design of the experiments and participated in editing the final copy of the manuscript. ASR was the overall project leader, participated in the design and coordination of the project and wrote the manuscript. All authors have read and approved the final manuscript.

## Supplementary Material

Additional file 1 **Alignment of the putative amino acid sequences deduced from the nucleotide sequences of the β-giardin gene of *Giardia lamblia *WB isolate [GDB: GL4812] and those of the β-giardin gene of *Giardia lamblia *GS isolate [GDB: GL2741]**. Click here for file
