# Supplementary material for: Immunodominant proteins α-1 giardin and β-giardin are expressed in both assemblages A and B of Giardia lamblia
Source: BMC Microbiol. 2011 Oct 19;11:233. doi: 10.1186/1471-2180-11-233 (PMC3206439; doi:10.1186/1471-2180-11-233)
Supplement: Additional file 1 — Alignment of the putative amino acid sequences deduced from the nucleotide sequences of the β-giardin gene of Giardia lamblia WB isolate [GDB: GL4812] and those of the β-giardin gene of Giardia lamblia GS isolate [GDB: GL2741]. [file 1471-2180-11-233-S1.DOC]

CLUSTAL 2.0.12 multiple sequence alignment

GL50803_4812 MSMFTSTRTLTQTMDKPDDLTRSATETAVKLSNMNQRVSRFHDKMENEIEVRRVDDDTRV 60

GL50581_2741 MSMFTSTRTLTQTMDKPDDLTRSATETAVKLSNMNQRVSRFHDKMENEIEVRRVDDDTRV 60

************************************************************

GL50803_4812 KMIKDAIAHLDRLIQTESRKRQASFEDIREEVKKSADNMYLTIKEEIDTMAANFRKSLAE 120

GL50581_2741 KMIKDAIAHLDRLIQTESRKRQASFEDIREEVKKSADNMYLTIKEEIDTMAANFRKSLAE 120

************************************************************

GL50803_4812 MGDTLNNVETNLQNQIAIHNDAIAALRKEALKSLNDLETGIATENAERKKMYDQLNEKVA 180

GL50581_2741 MGDTLNNVETNLQNQIAIHNDAIAALRKEALKSLNDLETGIATENAERKKMYDQLNEKVA 180

************************************************************

GL50803_4812 EGFARISAAIEKETIARERAVSAATTEALTNTKLVEKCVNEQLENVASEIRAIQEEIDRE 240

GL50581_2741 EGFARISAAIEKETIARERAVSAATTEALTNTKLVEKCVNEQLENVASEIRAIQEEIDRE 240

************************************************************

GL50803_4812 KAERKEAEDKIVNTLEDVVSKIQGGLSMVTKH 272

GL50581_2741 KAERKEAEDKIVNTLEDVVSKIQGGLSMVTKH 272

********************************

"*" indicates that the residues or nucleotides in that column are identical in all sequences in the alignment.
